# The Myeloid Receptor PILRβ Mediates the Balance of Inflammatory Responses through Regulation of IL-27 Production

**DOI:** 10.1371/journal.pone.0031680

**Published:** 2012-03-27

**Authors:** Cristina M. Tato, Barbara Joyce-Shaikh, Antara Banerjee, Yi Chen, Manjiri Sathe, Sarah E. Ewald, Man-Ru Liu, Daniel Gorman, Terrill K. McClanahan, Joseph H. Phillips, Paul G. Heyworth, Daniel J. Cua

**Affiliations:** 1 Merck Research Laboratories, Palo Alto, California, United States of America; 2 Department of Microbiology and Immunology, School of Medicine, Stanford University, Stanford, California, United States of America; 3 Institute for Immunity, Transplantation and Infection, School of Medicine, Stanford University, Stanford, California, United States of America; University of Nebraska Medical Center, United States of America

## Abstract

Paired immunoglobulin-like receptors beta, PILRβ, and alpha, PILRα, are related to the Siglec family of receptors and are expressed primarily on cells of the myeloid lineage. PILRβ is a DAP12 binding partner expressed on both human and mouse myeloid cells. The potential ligand, CD99, is found on many cell types, such as epithelial cells where it plays a role in migration of immune cells to sites of inflammation. *Pilrb* deficient mice were challenged with the parasite *Toxoplasma gondii* in two different models of infection induced inflammation; one involving the establishment of chronic encephalitis and a second mimicking inflammatory bowel disease in order to understand the potential role of this receptor in persistent inflammatory responses. It was found that in the absence of activating signals from PILRβ, antigen-presenting cells (APCs) produced increased amounts of IL-27, p28 and promoted IL-10 production in effector T cells. The sustained production of IL-27 led ultimately to enhanced survival after challenge due to dampened immune pathology in the gut. Similar protection was also observed in the CNS during chronic *T. gondii* infection after i.p. challenge again providing evidence that PILRβ is important for regulating aberrant inflammatory responses.

## Introduction

Dap12 is a modulator of the amplitude of an immune response, but in a very cell specific way [1]. There are many receptors that pair with Dap12 whose expression patterns, in part help govern the ultimate consequence of Dap12 signaling. One receptor partner of Dap12 is the paired-immunoglobulin-like receptor-beta (PILRβ). PILRβ is a type I glycoprotein with a single extracellular Ig-like domain and a truncated cytoplasmic tail [2]. PILRβ does not contain its own activation motif, but is dependent on Dap12 for ITAM mediated signals. PILRα is the inhibitory form of the receptor containing an ITIM within its intracellular domain that is thought to bind SHP-1/2 upon phosphorylation [3] [2]. PILRα and PILRβ are mainly expressed on cells of the myeloid lineage in both human and mouse populations, with most cells displaying both isoforms of this receptor on their surface constitutively. However, there is some differential expression between the paired receptors, with PILRβ as the primary isoform displayed by NK cells [4]. Expression of both receptors has also been shown to be upregulated on CNS-infiltrating macrophages and microglial cells during experimental autoimmune encephalomyelitis (EAE) (Joyce-Shaikh and Cua, unpublished data).

While expression of this paired receptor is relatively restricted, its potential ligands have a considerably more ubiquitous expression pattern. A CD99-like molecule was first identified on T cells as interacting with PILRβ on NK cells and activating their cytotoxic capacity. [4]. The same group also found that the CD99-like molecule was able to activate bone-marrow derived dendritic cells (DCs) and promote TNFα production. Although the PILRs contain a conserved arginine residue typical of Siglec family members, they do not themselves bind simple sialylated sugars. Instead, the ability of CD99 to bind PILRα and PILRβ was characterized as involving recognition of two O-glycosylated sites on CD99 and these sites are thought to be essential for stable interaction between receptor and ligand [5], [6]. Despite the efficiency with which the activating receptor is triggered by CD99 and CD99-like molecules, it was discovered that the affinity of PILRα for CD99 binding is significantly higher than the affinity of PILRβ [5].

CD99 is a glycoprotein that is thought to modulate a number of immune responses related to inflammation. It is expressed on the surface of a large variety of immune cells and tissues including activated T cells [7]. It may also play a role in regulating the migration of cells into tissues as it is also found on endothelial tight junctions where it has been shown to mediate immune cell extravasation from the blood [8], [9], [10]. Additionally, the inhibitory receptor, PILRα, is also thought to act as a co-receptor for viral glycoprotein B allowing pathogen entry into the host cell and in some cases viral persistence [11], [12]. There is an ever growing body of evidence to suggest that the CD99:PILRα/β interactions have a significant affect on the quality of the innate immune response. However, little is actually known about the overall function of these receptors or of their possible cross-regulation of each other.

Many DAP12 associated receptors have been shown to play key roles in regulating immune responses of macrophages and DCs during inflammatory responses. We therefore, used activating receptor-deficient mice to study the relevance of PILRβ in immune function. Specifically, given the receptor/ligand pattern of expression, we decided to employ two models of infection-induced chronic inflammation by challenging mice via alternative routes of infection with the protozoan parasite *Toxoplasma gondii* (*T. gondii*). In this way, we were able to analyze how PILRβ deficiency may alter the function of inflammatory antigen presenting cells (APCs) in tissues commonly targeted during autoimmune inflammation; the CNS and the mucosa. Low dose intra-peritoneal challenge with *T. gondii* promotes a systemic Th1 response during the acute phase of infection that serves to clear the immediate infection, drives the parasite into latency, and allows for pathogen persistence in the host's CNS [13], [14], [15]. A susceptible host will eventually succumb to toxoplasmosis, a chronic brain inflammation that is the result of increasing recruitment of immune cells into the brain to keep the parasite from reactivating. Both infiltrating and CNS resident APCs are important for maintaining the balance of pro- and anti-inflammatory mediators during chronic infection [15], [16].

Via high dose per oral infection with *T. gondii*, we were able to establish a murine model of inflammatory bowel disease (IBD) to test the impact of abrogating the function of PILRβ [17]. This route of infection promotes a robust Th1-type response that precipitates an aggressive immune-mediated pathology, causing necrosis of ileal villi, and resulting in the ultimate demise of the host within 7–14 days after challenge [18]. This model of infection mimics other IBD models with regard to immunological mechanisms and histological changes. For example, it is known that CD4+ T cells play a critical role in mediating pathology after high-dose infection with *T. gondii*, and an aberrant response to commensal flora worsens outcome. Thus, the innate immune response greatly affects outcome and severity of disease by establishing and then continuing to promote inflammation well beyond the initiating injury.

Through the use of activating receptor deficient mice (*Pilrb−/−*) we now show that APCs may use this receptor pair to balance the production of pro- and anti-inflammatory cytokines during an immune response. In the absence of PILRβ, mice infected by either route exhibited enhanced survival after parasitic challenge, which was characterized by increased production of IL-27, but not IL-10. APCs from PILRβ−/− mice exhibit a more tolerogenic phenotype which likely promotes enhanced control of inflammatory effector T cells.

## Materials and Methods

### Mice

*Pilrb*−/− mice were derived as previously described [19] were bred and housed within micro isolator caging units on site at MRL. Mice were bred as homozygous knockouts and wild-type (WT) C57Bl/6 (Jackson Laboratories, Bar Harbor, ME) were used as age- and sex-matched controls in all experiments. All animal procedures were approved by the Institutional Animal Care and Use Committee of Merck Research Laboratories in accordance with guidelines of the Association for Assessment and Accreditation of Laboratory Animal Care.

### Parasites

For *in vivo* experiments, mice were inoculated either intra-peritoneally with 20 cysts or per orally with 80–100 cysts of the Me-49 strain of *T. gondii*. Brain homogenate from chronically infected CBA/CaJ mice (Jackson Labs) were used as the source of cyst preparations for inoculation.

### Cyst counts

Individual brains from chronically infected mice were processed in 3 ml of PBS using syringes and consecutive 18 G, 20 G and 22 G needles to create a homogenate. The number of cysts present in 30 µl of homogenate was used to determine the total number of cysts present within each brain.

### Histology

Dissected brain or ileum were fixed in 10%NBF before embedding in paraffin. Hematoxylin and eosin staining was performed on sections of tissue for analysis of pathology.

### Brain mononuclear cell isolation and culture

Brain mononuclear cells (BMNC) were isolated from the CNS by performing collagenase digestion and separating lymphocyte populations using a percoll gradient as previously described [20], [21]. Cells were plated at 2×10^5^ cells per well in complete RPMI (Life Technologies, Gaithersburg, MD) supplemented with 10% FCS (HyClone Laboratories, Logan, UT), 1% HEPES, 50 µM 2-mercaptoethanol, 1% sodium pyruvate, and penicillin and streptomycin for a final volume of 200 µl. Cultures were stimulated with either αCD3 at 5 µg/ml or soluble *Toxoplasma* antigen (STAg) at 20–25 µg/ml (generous gift of C.A. Hunter, University of Pennsylvania, Philadelphia, PA).

### Splenocyte and lymph node culture

Spleens or LNs were harvested and dissociated into single-cell suspensions in complete RPMI as above. Erythrocytes were depleted from splenocyte suspensions using RBC Lysing Buffer (Sigma, St. Louis, MO), and cells were washed in complete media. Cells were plated at either 4×10^5^ or 2×10^5^ cells per well for splenocytes or LNs, respectively, and for a final volume of 200 µl. Cells were cultured as above for analysis of cytokine production after 2–3 days of restimulation at 37°C.

### APC enrichment

Magnetic cell sorting was performed to enrich populations of splenic dendritic cells (CD11c (N418) microbeads) and macrophages (CD11b microbeads), respectively from mice 5 days post i.p. parasite or PBS challenge using the standard protocols from Miltenyi Biotec (Auburn, CA). Cells were sorted using an autoMacs Separator according to the standard protocol. Cell pellets were used for RNA isolation and analysis of gene transcripts by RT-PCR (see below).

### Cytokine protein quantification

Serum samples or supernatants from cell culture were analyzed for cytokine protein levels using either ELISA kits for IFNγ, IL-27p28, IL-12p70, NO (all from R&D,), IL-10 (Invitrogen,), or LincoPlex (LINCO Research) for TNFα.

### Vector construction

The p2øC31.RSV.hAAT.bpA plasmid was provided by Dr. Zhi-Ying Chen (Stanford University, Stanford, CA). The vector was modified and a unique 5′ PmeI and 3′ PacI restriction sites flanking hAAT was introduced to facilitate directional cloning of cDNA's. PCR amplification was performed to place 5′ PmeI and 3′ PacI cloning sites on the mEBI3, mp28 and linked mIL-27 cDNA's and these were ligated with the modified minicircle producing vector. All constructs were verified by restriction digestion and sequencing of the cloned insert and flanking region.

### Production of minicircle DNA

Mini-circle DNA was produced following the methods described with some minor modifications [22]. For overnight cultures 1 liter of Terrific broth containing 100 ìg/ml Ampicillin was inoculated and incubated 18 hrs shaking at 270 rpm. Following the minicircle production method cultures were precipitated and stored at −80 C. Endotoxin free Qiagen megaprep kits were used for DNA purification, with 120 ml volumes of solution P1, 2&3. Minicircle DNA was eluted from the column, isopropanol was added and stored at −20 C. DNA was precipitated by spinning at 12 K, 30′ at 4 C, rinsed with 70% ethanol, air dried and resuspended in 1 ml of endotoxin-free Tris EDTA. Minicircle DNA was dialyzed in Midi MWCO 3.5 kDa tubes overnight against Tris EDTA. Purified minicircle DNA was verified by restriction digestion and sequencing.

### Minicircle Delivery

Minicircles were administered using a hydrodynamics-based transfection procedure as described previously [23]. Briefly, 20 mg of minicircle DNA in 2 ml of Ringer's solution was administered via tail vein injection within 5–8 seconds. Systemic expression of specific transgenes was verified using ELISA to analyze peripheral blood at various time points.

### RNA isolation and real-time quantitative PCR

Total RNA was extracted from dissected tissue by homogenizing organs into RNA STAT-60 (Tel-Test) using a polytron homogenizer, and following manufacturer's instructions. After isopropanol precipitation, total RNA was re-extracted with phenol∶chloroform∶isoamyl alcohol (25∶24∶1) (Sigma-Aldrich) using phase-lock light tubes (Eppendorf). For cell pellets, RNA was isolated using the RNeasy method, according to the manufacturer's protocol (Qiagen). Total RNA was reverse-transcribed using QuantiTect (Qiagen) according to manufacturer's instructions. Primers were obtained commercially from Applied Biosystems. Real-time quantitative PCR was performed using an ABI 7300 or 7900 sequence detection system. The absence of genomic DNA contamination was confirmed using primers that recognize genomic region of the CD4 promoter. Quantities of transcripts encoding ubiquitin were measured in a separate reaction and used to normalize the data by the –Ct method [24].

#### Parasite burden analysis

For parasite burden analysis, mesenteric lymph nodes or spleen were harvested on D3 or D9 post infection and flash frozen. Tissues were homogenized in Trizol (Invitrogen) reagent to extract RNA followed by cDNA synthesis using Superscript III First-Strand Synthesis System (Invitrogen) according to manufacturer's instructions. Gene expression was monitored by real-time PCR using Taqman primer/probe sets (Applied Biosystems) for mouse β-actin and *Toxoplasma* Sag1. Data was collected on the Mx3000P Q PCR System (Agilent Technologies).

### Statistical Analysis

Student's t-test was performed for analysis of significance and all results are expressed as mean ± SEM, unless otherwise noted. *P* values are presented where statistical significance was found.

## Results

### PILRα/β modulate CNS inflammation during chronic infection after i.p. challenge with *T. gondii*

Data from previous experiments performed in our laboratory showed that mRNA expression of both PILRs was increased in CNS resident microglial cells 14 days after induction of experimental autoimmune encephalomyelitis (EAE), the mouse model of Multiple Sclerosis compared to expression at day 0 (**Fig. S1a**). Therefore, to test whether activating receptor deficient APCs were capable of modulating responses in an infection-induced model of CNS inflammation, C57BL/6 WT and gene deficient mice were infected intra-peritoneally (i.p.) with 20 cysts of the Me-49 strain of *T. gondii* and followed out for 100 days. We first determined if there was normal expression of PILRα in the absence of PILRβ compared to WT mice during toxoplasmosis. When brain mononuclear cells (BMNCs) were harvested from chronically infected mice 60 days after challenge, and analyzed by RT-PCR for receptor expression, both WT and *Pilrb−/−* mice exhibited similar levels of PILRα inhibitory receptor (**Fig. S1b**). As expected, the PILRβ-activating receptor mRNA was detected in the WT mice, but was absent in gene-deficient mice following parasite infection.

**Figure 1:**
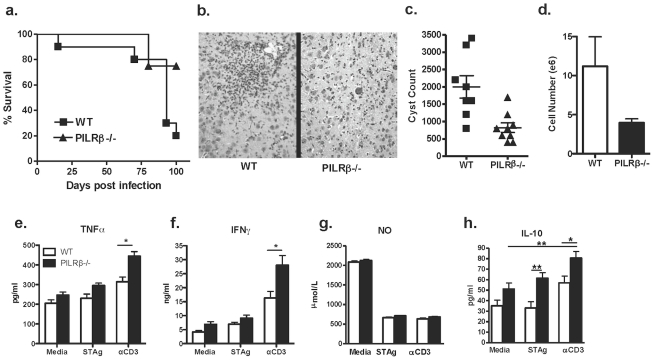
Characterization of the local inflammatory response during chronic i.p. infection. WT and *Pilrb* −/− mice were challenged i.p. with 20 cysts *T.gondii* and followed over time. Survival of WT and *Pilrb−/−* mice through 100 days post infection (a). H&E of brain sections reveal larger inflammatory foci in WT mice compared to *Pilrb−/−*, shown at 10× magnification (b). Total number of cysts present in the CNS of mice 60–90 days post infection (c). Actual numbers of BMNCs isolated from the CNS of mice (d). Recall assays using BMNCs isolated from WT and *Pilrb−/−* mice and cultured for 72 hrs in the presence of Media alone, STAg, or αCD3. Levels of protein are shown as detected by ELISA for TNFα (e), IFNγ (f), NO (g), and IL-10 (h). For panel a, n = 5–15 mice/group for each of 3 experiments performed. For panel c, pooled data from 2 experiments are shown, p<0.004. For panel d, one representative experiment of 2 is shown, p<0.05. For panels e,f, and h, pooled data from 2–3 experiments are shown, *p<0.005; **p<0.002.

When compared for survival, the WT C57BL/6 mice—which are susceptible to infection—began succumbing between 60 and 90 days post-challenge, whereas *Pilrb* −/− mice remained resistant beyond 100 days post infection (**Fig. 1a**). In addition, histological examination of the brain from chronically infected mice confirmed the presence of more inflammatory infiltrate in WT compared to *Pilrb* −/− brain, suggesting that the enhanced resistance in *Pilrb−/−* mice is due to reduced immunopathology associated with fewer CNS infiltrating cells (**Fig. 1b**).

In order to better characterize the mechanism behind this enhanced survival, either whole brain or BMNCs were isolated from both groups after 60 days of infection and parasite burden and inflammatory infiltrate were quantified. Cyst counts revealed significantly lower pathogen load in the activating receptor *Pilrb−/−* mice compared to WT controls (**Fig. 1c**). When BMNCs isolated from chronically infected mice were examined by flow cytometry, no difference in types of infiltrating cells, or percentages of cell population were observed between strains (data not shown). However, when actual numbers of infiltrating cells were determined, we found that *Pilrb−/−* mice had significantly fewer numbers of BMNCs corresponding to enhanced resistance to encephalitis (**Fig. 1d**).

Chronic resistance to toxoplasmosis requires a delicate balance between pro-inflammatory cytokines like IFNγ and TNFα to control infection and regulatory cytokines like IL-10 to prevent immunopathology [25]. Therefore, we isolated BMNCs from chronically infected mice and determined if there was a difference in production of cytokines. In response to αCD3 restimulation there was significantly more TNFα and IFNγ protein detected in the supernatants of *Pilrb* −/− cultures compared to WT cultures (**Fig. 1e and f**). One would have expected more inflammatory cytokine production in WT cultures given that WT mice have a higher parasite burden than *Pilrb* −/−mice at the same time-point. More IFNγ production in *Pilrb* −/− mice would also suggest the presence of more inflammatory cells, however, our previous data revealed fewer cells infiltrating the CNS. One explanation for this paradox is that the enhanced IFNγ production in the *Pilrb−/−* may lead to better control of parasite reactivation, and thus better control of parasite numbers. Nitric oxide production in the CNS is an important mechanism of pathogen control during chronic infection, however when nitric oxide levels were analyzed in BMNC cultures, no difference was seen under any condition (**Fig. 1g**). Higher levels of inflammatory cytokine production would need to be balanced by regulatory cytokines in order to keep CNS inflammation subdued. Previously published studies have assigned IL-10 an important role in controlling CNS inflammation during chronic toxoplasmosis [15], [26], [27]. When IL-10 production was assessed in BMNC cultures, significantly greater levels were detected in *Pilrb* −/− BMNC cultures not only in response to αCD3, but also after antigen restimulation over amounts produced in WT cultures (**Fig. 1h**). These data again suggested that *Pilrb−/−* APCs promote IL-10 secreting T effector cells that home to the CNS and provided a more stringent regulation of inflammation in the CNS. However, the enhanced production of anti-inflammatory cytokines did not preclude the possibility that *Pilrb* −/− mice may also have an increased ability to clear the parasite from the local site of infection during the acute response.

### Increased systemic IL-27p28, but decreased IFNγ in *Pilrb* −/− mice after i.p. infection

**Figure 2:**
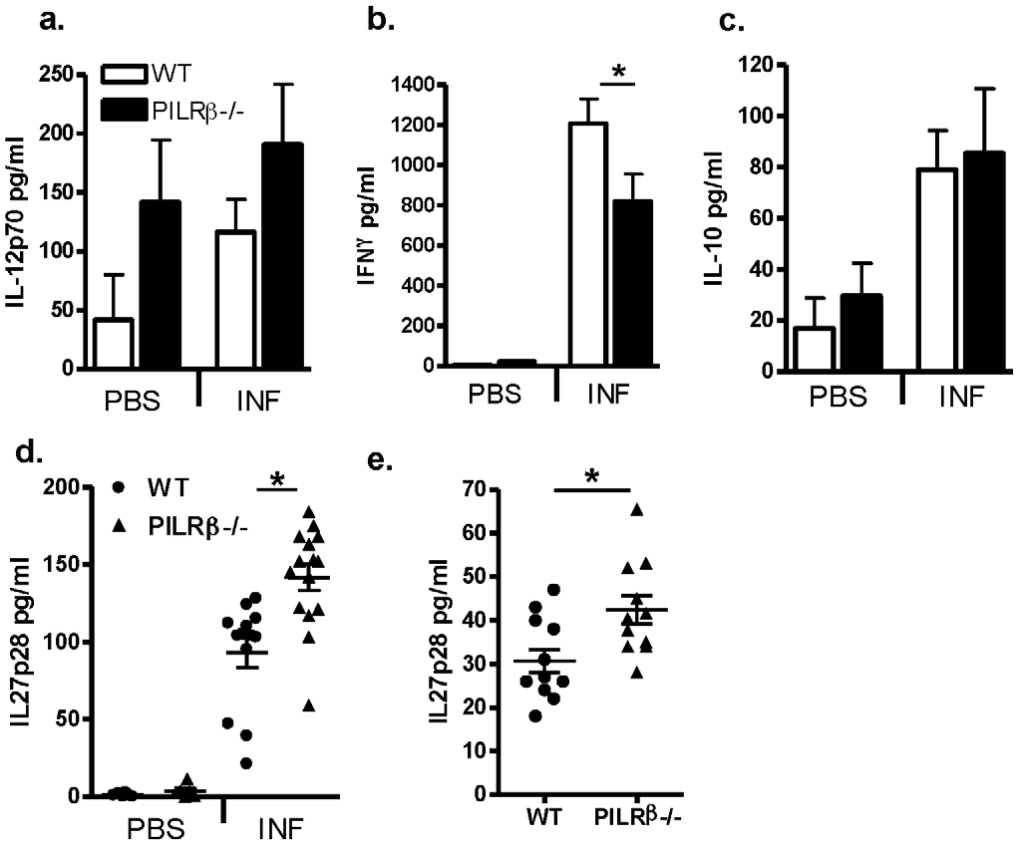
Systemic cytokine response in WT and *Pilrb* −/− mice after acute and chronic i.p infection. WT and *Pilrb* −/− mice were challenged i.p. with either PBS or *T.gondii* and serum cytokine protein levels were determined by ELISA during acute infection; IL-12p70 (a), IFNγ (b), IL-10 (c), and IL-27p28 (d) all on day 5. Alternatively, IL-27p28 was analyzed on day 60–90 after challenge (e). Pooled data from 2–3 experiments is shown for all graphs. For panel b, p<0.043; for panel d, p = 0.0007 and for panel e, p<0.01.

In order to characterize the acute immune response to infection with *T. gondii*, both WT and *Pilrb* deficient mice were infected i.p. as before and lymphocyte responses were analyzed at various timepoints during the first two weeks post challenge. When serum from day 5-infected mice were tested for inflammatory cytokines, systemic IL-12 production was similar. However, circulating IFNγ was reduced in *Pilrb*−/− mice (**Fig. 2a and b**). Although serum IL-10 was enhanced in both WT and *Pilrb* −/− mice after infection, there was no difference between strains (**Fig. 2c**). Interestingly, the reduction in IFNγ production did not have a deleterious effect on controlling infection, because analysis of day 3- and day 5-peritoneal exudate cells (PECs) revealed no differences in parasite burden between strains (**Fig. S2a**). Since there were no obvious differences in the acute phase activation of the immune response to *T. gondii*, we wondered whether there might be differences in other regulatory cytokines at this early timepoint.

It has been recently shown that the presence of IL-27 can directly induce a strong regulatory effect on CD4 T cell production of proinflammatory cytokines, an effect that can also be mediated by enhancing IL-10 production [28], [29], [30] [31] [32]. Specifically in the context of infection with *T. gondii*, IL-27 has been shown to play a significant role in regulating Th1 activation and preventing immunopathology (reference 30). Given that effector T cells from *Pilrb−/−* mice have an increased propensity for secreting IL-10, we assessed the production of IL-27 in WT and *Pilrb* deficient mice after i.p infection as a potential mechanism for the enhanced resistance to toxoplasmosis. Thus, IL-27 was analyzed in the serum of both acute and chronically infected mice after infection. Interleukin-27 is not constitutively expressed, as no detectable amount of IL-27p28 was observed in the serum of either WT or *Pilrb−/−* mice injected with PBS alone. While levels of IL-27p28 were increased in both strains by 5 days post infection, there were significantly higher levels present in serum from *Pilrb* −/− mice than from WT mice (**Fig. 2d**). Interestingly, systemic production of IL-27p28 did not lead to a corresponding increase in IL-10 protein at this early day 5 time point, as there was no detectable serum IL-10 in either strain (data not shown). Additionally, production of IL-27p28 was found to be maintained throughout the course of infection in both WT and KO mice, even at day 60 post infection (**Fig. 2e**). Once again significantly higher levels of p28 were found in the serum of chronically infected *Pilrb* −/− mice compared to WT mice. It may be predicted that the p28 expression that is present early during infection could enhance the early Th1 response in *Pilrb* −/− mice and allow for more efficient clearance of the parasite. Thus, there would be less of a requirement for sustained IFNγ expression under such conditions.

### *Pilrb* −/− survive lethal high dose infection with *T. gondii*

**Figure 3:**
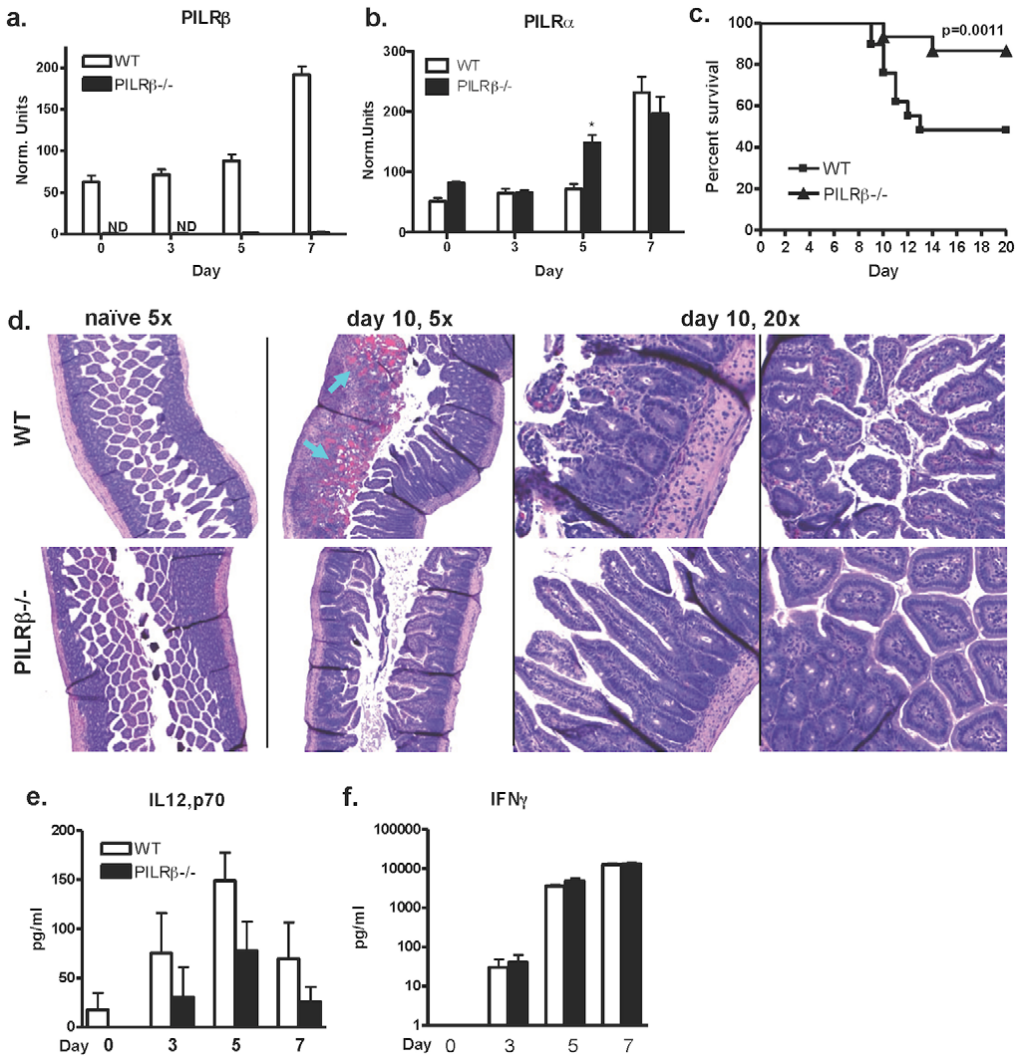
Normal systemic response to infection, but enhanced resistance to high dose per oral infection. Expression of *Pilrb* (a) and *Pilra*(b) mRNA in the ileum of infected WT and *Pilrb* −/− mice at various timepoints after infection. Survival of WT and *Pilrb−/−* mice after high dose oral infection, n = 8–11 mice/group for each of 3 experiments performed (c). H&E of ileum sections from WT (top panels) and *Pilrb−/−* (lower panels), at day 0 (5×) or day 10 post-peroral challenge 5× and 20× magnification, respectively. Arrows indicate the presence of blood (d). Levels of cytokine protein present in the serum of naïve or infected mice at various timepoints, IL-12p70 (e) and IFNγ (f).

Another model in which IL-10 production is important in mediating resistance is an infection induced model of IBD, in which a lethal inflammatory response persists in the ileum after per oral administration of high dose *T. gondii* [33]. In order to test the impact of the absence of *Pilrb* on APCs under these conditions, mice were per orally challenged with 80–100 cysts of Me-49 and survival was monitored. To establish whether or not the PILRs were expressed in the mucosa during infection, sections of terminal ileum from infected mice were analyzed by RT-PCR to track the expression of PILRα and PILRβ over time. Messenger RNA for both the inhibitory and activating forms of the receptor were found to increase significantly in the gut of WT mice by day 7 post infection (**Fig. 3a, b**). As expected, no *Pilrb* expression was detectable in gene deficient mice, however there was greater *Pilra* expression observed in the ileum of *Pilrb* −/− mice compared to that of WT mice by day 5 (**Fig. 3b**). These data suggest that there is an earlier upregulation of the inhibitory receptor in the absence of its activating partner. Furthermore, it was found that WT mice succumbed to infection within 14 days of challenge with only 48% survival on average, *Pilrb* −/− mice survived significantly longer, with 87% survival (**Fig. 3c**).

Early differences in parasite burden may have an effect on the magnitude of inflammation that is established gut. In order to determine if there were differences in parasite load after high-dose infection, we used qPCR to quantitate the amount of parasite DNA in the mesenteric lymph nodes (MLN) and spleen 3 and 9 days post-infection, respectively. Again, we found no difference in the MLN at the earliest timepoint of 3 days after infection (**Fig. S2b**). Later by day 9, however, *Pilrb*−/− spleens show decreased parasite burden compared to WT spleens and this is consistent with what we observed during chronic i.p. infection (**Fig. 2b**). Locally, inflammation can also be regulated by cellular recruitment to the tissue and the resultant milieu of cytokines. To determine whether PILRα/β signaling can indeed affect either of these aspects of the immune response, histological analysis of ileum from both WT and *Pilrb* −/− mice after infection was performed. Overall, WT sections of ileum revealed the presence of more cellular infiltrate in the mucosa and more blood compared to *Pilrb* deficient sections 10 days after challenge (**Fig. 3d**). When serum levels of cytokine were analyzed at regular intervals during the first 7 days of infection there were, surprisingly, no significant differences in systemic IL12p70 or IFNγ production between strains at any time point (**Fig. 3e, and f**).

### Enhanced host survival of *Pilrb* −/− to lethal infection is associated with increases in IL-27p28

**Figure 4:**
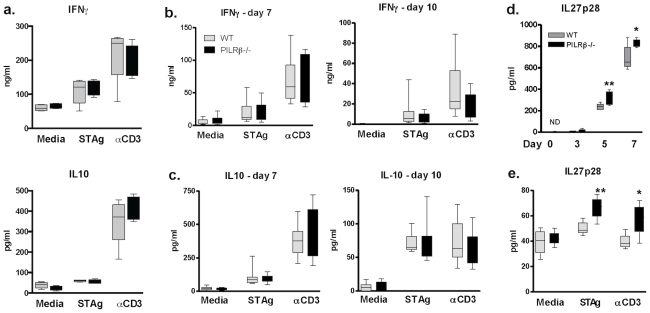
Host survival is characterized by increased capacity to produce IL-27p28. Recall assays using MLNs at day 5 (a); and splenocytes at day 7 (b and c, left panels) or day 10 (b and c, right panels) after peroral challenge. Amount of IFNγ (a, top graph; and b, left and right) or IL-10 (a, bottom graph; and c, left and right) detectable in supernatants of WT (grey bars) and *Pilrb−/−* (black bars) after 3 days of culture in media alone, with STAg, or with αCD3. For panel a, one representative experiment of two shown; for panels b and c, combined data from 3 independent experiments shown. Time course of IL-27p28 protein in serum from infected WT (grey bars) and *Pilrb* −/− (black bars) mice as detected by ELISA, mean ± SD is shown (d). Values between strains on days 5, p = 0.0219 and 7, p = 0.0318 are all significantly different. Recall assay using MLNs at day 5 post-peroral infection and cultured as above. Amount of IL-27p28 detectable in supernatants after 3 days of culture (e). Mean ± SD are shown. For STAg, p = 0.0143; for αCD3, p = 0.0371.

To examine the function of effector cells after infection, MLNs and spleens were harvested and cultured in recall assays at days 5 or days 7 and 10 post-infection, respectively, and supernatants were analyzed for the presence of cytokine. Neither IFNγ nor IL-10 production by T cells was found to be significantly different between cultured WT and *Pilrb* deficient MLNs (**Fig. 4a**) or splenocytes in response to soluble Toxoplasma antigen (STAg) or αCD3 restimulation at any timepoint (**Fig. 4b and c**).

Thus, our data suggests that the resistance of *Pilrb−/−* mice to intestinal necrosis during high dose *T. gondii* infection is not due to a consistently observed alteration of T cell effector cytokines during inflammation. Importantly, since *Pilrb* is not expressed on T cells, the mechanism by which this receptor modulates inflammation may not be T cell specific. It was therefore proposed that there must be a change in the quality of APC activation either because of altered cytokine production and/or a cell contact-dependent mechanism.

We next analyzed the production of IL-27, both systemically and after antigen recall. We found that serum IL-27p28 levels were increased by day 5 and 7 in the *Pilrb* −/− mice compared to WT (**Fig. 4d**). In order to further characterize the cytokine milieu present during early T cell activation, IL-27p28 production was measured in MLN cultures after 5 days of infection. In response to both antigen or αCD3 stimulation IL-27p28 was again found to be increased in the absence of *Pilrb* compared to WT cells (**Fig. 4e**). Taken together these data suggested that in the absence of *Pilrb*, enhanced production of IL27p28 may likely lead to a more efficient control of inflammatory T cells and increased host survival to lethal immune-pathology. Therefore, in the absence of receptor activation, APCs may become more tolerogenic in phenotype and promote a tighter regulation of the resultant inflammatory response.

### Splenic DCs exhibit a more “tolerogenic” phenotype in the absence of PILRβ signaling

**Figure 5:**
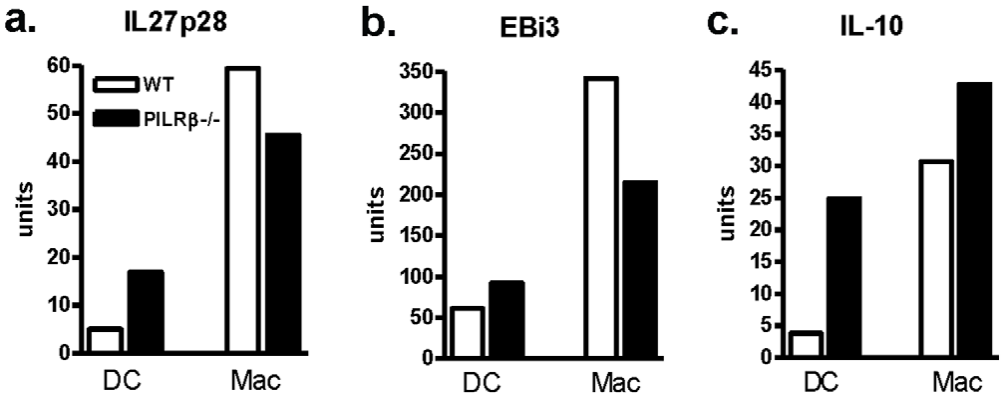
Cytokine mRNA expression in dendritic cells and macrophages from infected mice. Populations of DCs and macrophages were enriched from the spleens of WT and *Pilrb* −/− mice 5 days after i.p. challenge. Cells were then analyzed by RT-PCR for mRNA levels of IL27p28 (a) EBi3 (b) and IL-10 (c). Pooled data from one of two representative experiments is shown.

Since PILRβ is expressed on many myeloid subsets, we undertook experiments to identify which cells were the main source of IL-27p28 in our model. RT PCR of splenic APC populations isolated after i.p. parasitic challenge, revealed increased p28 mRNA in DCs from *Pilrb−/−* mice compared to WT mice, but no difference in EBi3 message (**Fig. 5a,b**). In contrast, macrophages from gene deficient mice exhibited lower message levels for both IL-27 sub-units compared to those isolated from WT mice. We also observed increased mRNA for IL-10 in *Pilrb−/−* DCs compared to WT populations (**Fig. 5c**), however, there were less consistent levels of IL-10 message present in macrophage populations. Similar levels of PILRα message were present in both WT and *Pilrb*−/− strains for each APC population making it unlikely that a compensatory upregulation of the inhibitory receptor had occurred in the absence of the activating receptor for either DCs or macrophages (**Fig. S3**). Thus, these results suggest that by producing higher levels of IL-27, the DC population may be able to influence the strength of the inflammatory response despite high levels of inflammatory cytokines. These data reveal that when PILRβ signaling is abrogated, DCs and possibly macrophages may become more tolerogenic in phenotype by producing significantly more IL-27p28 and in turn are better able to influence the overall potency of the inflammatory response.

### IL-27 and not p28 alone is required to mediate resistance to challenge with *T. gondii*

**Figure 6:**
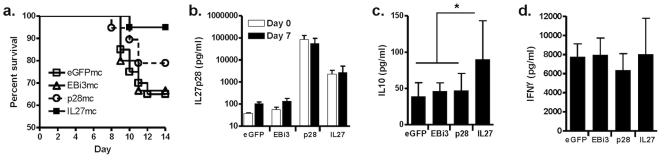
IL-27 can mediate resistance to parasite induced immunopathology. Expression of p28, EBi3, IL27 hyperkine, or GFP control were induced in vivo using minicircle DNA for systemic expression prior to high dose peroral infection with *T. gondii*. Survival curve of all groups 14 days post infection (a), p<0.02 for IL-27 vs. eGFP treatment groups. Serum cytokine levels for each treatment group at day 0 (white bars) and day 7 (black bars) post infection showing IL-27p28 (b), IL-10 (c), p<0.004, and IFNγ (d). For all panels means ± SD are shown. For panel a, n = 5 or 10 mice/group for each of 3 experiments performed. For panels b, c and d, n = 10 mice/group for the experiment shown, 2 total experiments performed.

Because it is only possible to detect p28 protein in serum and supernatant samples, we tested if it was indeed IL-27, and not p28 alone that could mediate enhanced resistance to infection in the *Pilrb−/−* mice. We injected DNA plasmids to induce over expression of either p28, EBi3 or a linked hyperkine form of IL-27 protein in C57Bl/6 mice before high dose per oral pathogen challenge. When mice were monitored for survival it was found that deaths occurred in all control groups beginning at day 8 post-infection, but not in the IL-27-expressing group, suggesting a direct role for IL-27 in mediating resistance (**Fig. 6a**). When serum levels of p28 were tracked, it was found that p28 was expressed over a log greater compared to the p28 protein in the IL-27-expressing group. Notably, the levels of p28 protein in the IL-27-expressing group were significantly higher compared to control groups (**Fig. 6b**). Interestingly, in the presence of robust IL-27 expression we also observed a greater induction of IL-10 in the serum of IL-27-expressing mice by day 7 post-infection compared to control or p28-expressing mice (**Fig. 6c**), while systemic IFNγ protein was similar between all groups (**Fig. 6d**). Taken together these data provide evidence for a direct link between IL-27, the promotion of systemic IL-10 and the enhanced survival of mice after challenge.

## Discussion

DAP12 has been shown extensively to regulate the inflammatory responses of innate immune cells (reference 1). Like others, we have shown here that interruption of DAP12 signaling results in the increased production of both IFNγ and IL-10 and decreased activation of both macrophages and DCs [34]. However, our observations are unique in that for our system the immune response is significantly altered with the elimination of only one DAP12 partner, through the deletion of PILRβ, keeping all other signaling pathways in tact. Thus, the effect we have observed may impact more specifically on alternative mechanisms that have been recently shown to regulate immune responses, such as the downstream effects of IL-27.

Previously published work has identified IL-27 as having a significant influence on the balance between an effective immune response and curbing of immune pathology [35]. IL-27 produced by myeloid cells has been shown not only to promote Th1 responses during an acute response to infection, but also to be required for damping of effector cell responses during the late adaptive stage of infection once the pathogen is controlled [29], [36], [37], [38]. The pleiotropic effects of IL-27 are also evident in the data presented here. Despite a decrease in systemic IFNγ production after i.p. challenge, *Pilrb* deficient mice had no issue clearing the parasite from the local site of infection. Importantly, systemic IL-10 production was not different at early time points, but comes up later in the response and is likely derived from T-cells. When activated T cells were restimulated with either antigen or αCD3, they were capable of normal IFNγ as well as IL-10 production. This combination provides for adequate protection against the parasite during acute infection while sustaining tight regulation of the chronic inflammatory response in the CNS. We have shown that PILRα and PILRβ are expressed not only on inflammatory macrophages and DCs, but also CNS resident cells such as microglial cells. Interestingly, it is already known that cells native to the CNS, such as microglia and astrocytes produce IL-27 under some conditions of inflammation [39], [40], [41]. These expression data, along with our observations that the systemic responses are not greatly altered in *Pilrb−/−* mice after challenge, may suggest that PILRα and PILRβ primarily play a role in regulating local inflammatory responses.

To test this idea we compared survival of WT and *Pilrb* −/− mice after high dose per oral infection, where better control of local inflammation would be beneficial. Susceptibility in this case is the result of immune mediated destruction of the mucosa by IFNγ-secreting CD4+ T cells and is independent of systemic infection [42], [43]. Again, while the acute, systemic response to high dose infection was not altered, the data we have presented here suggest that the absence of *Pilrb* significantly changes the local inflammation in the gut and allows for decreased epithelial damage and enhanced survival that is directly mediated by IL-27. Furthermore, we show specifically that DCs deficient for the activating receptor PILRβ express more IL-27 message and exhibit a more regulatory phenotype with the capacity for efficient damping of the T effector cell response.

Effector T cell responses to *T. gondii* have been shown to involve the generation of IFNγ/IL-10 double positive cells that are required for preventing the immunopathology in response to the parasite [44]. As previously discussed, IL-10 production is vitally important for a limited mechanism of resistance after high-dose per oral challenge with *T. gondii*, similar to its' regulatory role in IBD. Furthermore, there is an ever-increasing body of literature showing IL-27-dependent upregulation of IL-10 as a mechanism for immune regulation. Thus, while we now show that IL-27 secretion by APCs can be modulated through ligation of the PILRα and PILRβ receptors, we cannot eliminate the possibility that IL-10 may also contribute to the increased survival we observed after peroral infection.

In a normal host, initial ligation of the more highly expressed PILRβ leads to activation of APCs as they migrate into target tissues during the early inflammatory response. This first interaction promotes a proinflammatory milieu due in part to high levels of IL-12 secretion and lower levels of IL-27. As APCs become activated, they are able to increase the amount of inhibitory PILRα on their surface, and with its higher affinity for CD99-like molecules, may then out-compete the activating receptor for ligand. This change may cause a corresponding change in levels of IL-27 production by APCs, and possibly a corresponding increase in IL-10 production by activated T cells at the site of inflammation which together serve to down-regulate the inflammatory response. Thus, PILRα provides a negative feedback loop for the activating effects of PILRβ.

Related to its suppressive affects on Th17 cells it has been recently shown that IL-27 can ameliorate inflammation and pathology associated with experimental autoimmune encephalomyelitis (EAE) and collagen induced arthritis (CIA), murine models of multiple sclerosis and rheumatoid arthritis [45], [46], [47], [48]. Thus, we have provided evidence for the first time, that the PILRα/β receptor pair may directly promote a more tolerogenic phenotype and be a possible target for altering the local proinflammatory environment in both the mucosa and CNS.

## Supporting Information

Figure S1 **Expression as determined by RT-PCR, of** ***Pilra*** **(a, left panel) and** ***Pilrb*** **(a, right panel) in microglial cells 14 days after induction of EAE or in BMNCs from mice infected with** ***T. gondii*** **for 60–90 days (b, left and right panels, respectively).**
(TIFF)

Figure S2 **Parasite burden after infection with** ***T. gondii*** **.** Percentage of infected cells in the PECs of WT and *Pilrb*−/− mice 5 days after i.p. challenge (a). *Toxoplasma* titer was monitored by transcript levels of SAG1 3 days post infection in the MLN at day 3 (b, left panel), and spleen at day 9 (b, right panel) from WT and *Pilrb*−/− mice after peroral challenge with a high-dose of *T. gondii*. For each organ SAG1 transcript was normalized to mouse β-actin and the mean and standard deviation of 3 or 4 mice per condition is shown. Day 3 p = 0.146; Day 9 *p = 0.042.
(TIFF)

Figure S3 **Inhibitory receptor,** ***Pilra*** **mRNA expression in dendritic cells and macrophages.** Populations of DCs and macrophages were enriched from the spleens of WT (open bars) and *Pilrb* −/− mice (black bars) 5 days after i.p. challenge. Cells were then analyzed by RT-PCR.
(TIFF)
