# Supplementary figures and images for: The Myeloid Receptor PILRβ Mediates the Balance of Inflammatory Responses through Regulation of IL-27 Production
Source: PLoS One. 2012 Mar 27;7(3):e31680. doi: 10.1371/journal.pone.0031680 (PMC3313972; doi:10.1371/journal.pone.0031680)

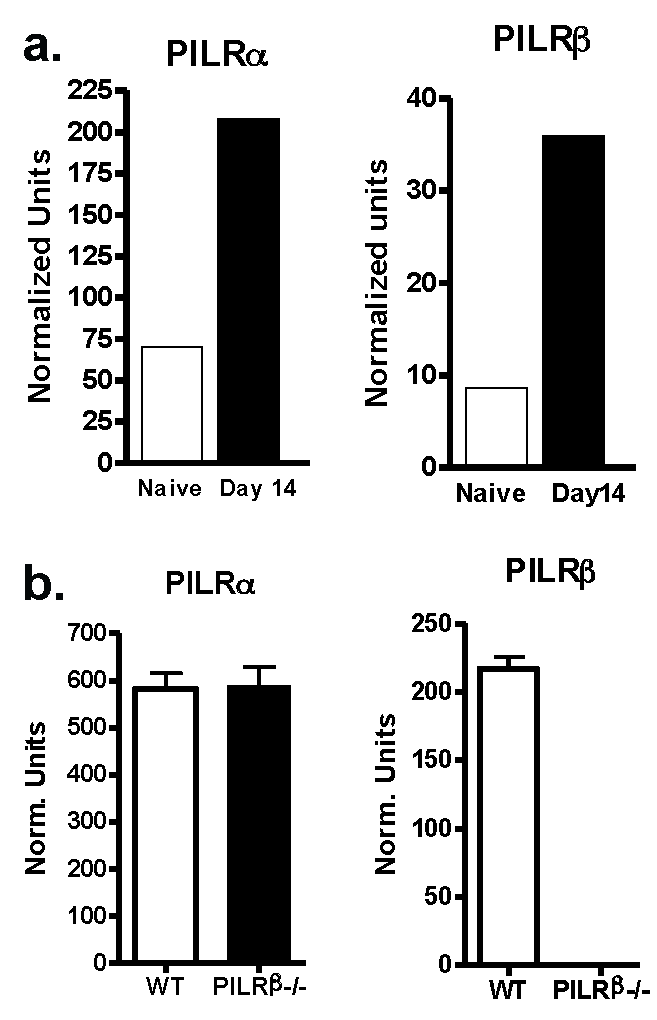

Supplement: Figure S1 — Expression as determined by RT-PCR, of Pilra (a, left panel) and Pilrb (a, right panel) in microglial cells 14 days after induction of EAE or in BMNCs from mice infected with T. gondii for 60–90 days (b, left and right panels, respectively). (TIFF) [file pone.0031680.s001.tif]

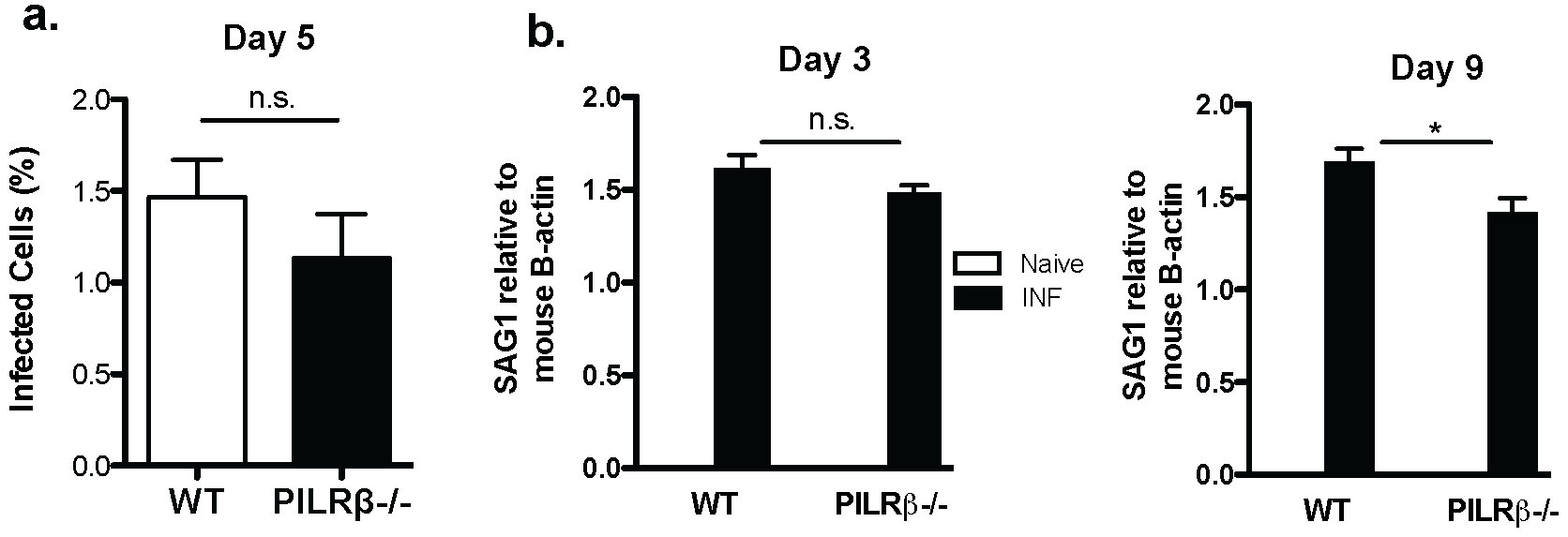

Supplement: Figure S2 — Parasite burden after infection with T. gondii . Percentage of infected cells in the PECs of WT and Pilrb−/− mice 5 days after i.p. challenge (a). Toxoplasma titer was monitored by transcript levels of SAG1 3 days post infection in the MLN at day 3 (b, left panel), and spleen at day 9 (b, right panel) from WT and Pilrb−/− mice after peroral challenge with a high-dose of T. gondii. For each organ SAG1 transcript was normalized to mouse β-actin and the mean and standard deviation of 3 or 4 mice per condition is shown. Day 3 p = 0.146; Day 9 *p = 0.042. (TIFF) [file pone.0031680.s002.tif]

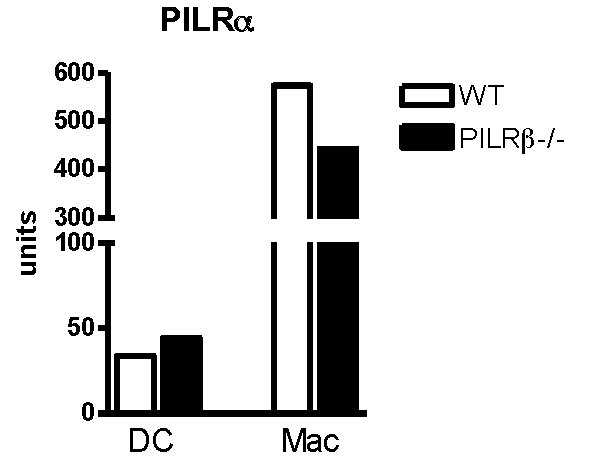

Supplement: Figure S3 — Inhibitory receptor, Pilra mRNA expression in dendritic cells and macrophages. Populations of DCs and macrophages were enriched from the spleens of WT (open bars) and Pilrb −/− mice (black bars) 5 days after i.p. challenge. Cells were then analyzed by RT-PCR. (TIFF) [file pone.0031680.s003.tif]
